# Genital Irritation, Together with Some Remarks on the Hygiene of the Genital Organs in Young Children

**Published:** 1880-12

**Authors:** Roswell Park

**Affiliations:** Demonstrator of Anatomy, Chicago Medical College, etc.; 1558 Wabash Ave.


### CHICAGO MEDICAL JOURNAL & EXAMINER.

Vol. XLI.—DECEMBER, 1880.—No. 6.

### Original Communications.

### Article I.

### Genital Irritation, together with some Remarks on the Hygiene of the Genital Organs in Young Children. By Roswell Park, a.m., m.d., Demonstrator of Anatomy, Chicago Medical College, etc. (Read before the Chicago Medical Society, October 18, 1880.)

A few years ago the interest of the general practitioner was aroused by brilliant reports of cases of young children in whom a variety of disorders, apparently of the nervous system, had been diagnosed as reflex phenomena caused by irritation of the genital organs, and which were speedily relieved by operative measures, usually either circumcision or clitoridectomy.

Dr. Sayre, of New York, has done, perhaps, as much as any one to call attention to the advisability of a careful scrutiny as to the condition of the *pars genitalia* of infants and children committed to our professional care. This scrutiny should not cease with a simple examination to see whether there be any phymosis or other abnormality, but, when possible, should be made the occasion for a brief but forcible epitome of an extended lecture upon the advantages of, and necessity for, strict cleanliness and common-sense care of the organs. Explicit instructions and even demonstration are required. The amount of ignorance displayed by the average father and mother as to the proper hygeine of the genito-urinary organs of their children is simply astonishing; and the physician who has his patient's interests at heart must do more than simply hint that care and cleanliness are required; he must show exactly what is to be done and watch to be sure that it is done. These are really matters of vital interest, and there is no excuse for false delicacy.

Excluding consideration of vesical calculus, there are in young male children no lesions of the genito-urinary organs which cause such a varied assortment of reflex phenomena as those connected with the prepuce, and which may be mentioned as follows: phymosis pure and simple, preputial stenosis, partial or complete preputial adhesions, and retention of smegma. To this enumeration might be added complications of any or all of these conditions.

The prepuce is always relatively long at birth, but can usually be easily retracted. If retraction be practiced from the outset, and strict cleanliness be observed, all goes well. But let the nurse be ignorant and the medical attendant inattentive and adhesions gradually take place, while the smegma accumulates, and we have then a condition pretty certain to cause mischief *some* time unless remedied.

When the adhesions are quite complete the smegma, after a time, ceases to collect, and there may follow, as a result of pressure, a partial atrophy of the glands which secrete it; while the substance itself may possibly undergo a calcareous degeneration and be noticeable as a concretion beneath the prepuce. This condition, while very seldom met with here, is not infrequent in certain quarters of the globe. In cases where the adhesions are not so complete, or especially where there is such stenosis of the preputial orifice that there occurs a ballooning of the foreskin with each effort at micturition, the retained smegma is never allowed to harden, but is at short intervals macerated in urine, and finally, by its own accumulation and by the deposition of urinary salts, the preputial sac becomes filled up with a pultaceous debris which may extend it to a remarkable degree.

That such a state of affairs must beget deleterious results is evident. The disorder thus set up at one end of the urinary tract is not always limited to its place of origin, but may cause in succession a balanitis, urethritis and cystitis, until, at last, the kidneys are affected, and then, if not before, the constitution suffers severely. Dr. J. C. Hupp has reported (Trans. Med. Soc. W. Va., 1879) an interesting case of a patient who, at an advanced age, succumbed to a complication of these troubles resulting from a congenital phymosis.

It may be asked how it happens that we find very few cases of phymosis in adults when we have so large a proportion—as I shall show—among children? The answer is that a spirit of investigation or prurient curiosity leads most of our lads to discover that a phymosed condition of the prepuce is not a natural or conventional one, and that the discovery leads to efforts to correct it. Constant efforts to retract the prepuce amount to a rational, gradual dilatation, and, if persisted in, will usually prove successful. Children brought up in towns and cities, or who are sent to public or private schools, very soon either discover for themselves or are taught to recognize any little anomaly in this portion of their anatomy, and the ridicule to which this subjects them among their playfellows leads to arduous and sometimes injurious efforts to correct it. To such an extent is this true, that when I see a young man with a phymosis, I usually find that he was brought up apart from other children.

There cannot be the slightest doubt that the irritation of the retained smegma, of partial or complete adhesions, or of a contracted orifice to the foreskin, is the frequent inciting cause not only of the various nervous phenomena to which general attention has been drawn, but also of masturbation and some forms of hysteria. Indeed, more than one boy has confided to me that his habits of self-abuse dated from the time when he instituted efforts to make his prepuce resemble those of his comrades.

Another inquiry which suggests itself is this: If this condition of affairs is capable of engendering so much mischief, and if it is so common, why do we not hear of more cases of serious nervous disturbance caused by it? I would answer such a question by saying that, in the first place, I think that, as a correct explanation of some cases of nervous disorder in children, it is still overlooked; and, in the second place, that we are inclined to forget to what an extent the impressionability of different children may vary. If genital irritation may cause convulsions in one case, paralysis in a second, or club-foot in a third, it may also cause urticaria, or indigestion, or diarrhœa, or incontinence of urine in yet others. In fact, I shall briefly report two cases of chronic diarrhœa in little boys, which resisted all medication, but were speedily relieved by removing the irritating causes about the genitals. And in a number of cases I have broken up the disgusting habit of wetting the bed at night by freeing the glans from some adhesions between it and the prepuce.

Every recent writer has called attention to the prominence of complete or partial phymosis in the etiology of nocturnal incontinence, and an enthusiast, realizing how common the condition is, may be deluded into the idea that all he has to do to break up the habit at once is merely to release the foreskin by simple and appropriate measures. But, in the majority of cases, said enthusiast will be deceived, because he loses sight of the fact that the habit has become firmly established by the time he is called upon for advice, and by looking for immediate relief he displays an overweening impatience. Let the operation be performed by all means, if indicated, and then, if needed, belladonna and strychnia with other nerve tonics and sedatives may be used; and then, too, proper moral suasion may be resorted to, with prospect of success.

If, beside asking the above two questions, one shall persist still in pointing out numerous individuals who have reached adult life with a prepuce in one of the conditions described, and who have never suffered in consequence, and if he shall argue upon these premises that undue prominence is given to the subject, I can still reply that many—though perhaps not *as* many—go through life with much more serious congenital variations or anomalies whose existence is never heard from or detected; and that the mere fact so many do escape serious trouble does not necessarily imply that others may not suffer severely, as they unquestionably do.

I have not been able to find any literature which would give any idea as to the comparative immunity from preputial lesions which children may enjoy, or as to the percentage of cases in which we may expect to find some deviation. Having surgical supervision of an institution in which over one hundred children are cared for constantly, I concluded to investigate for myself. By the opportunity thus afforded me, and from my notes of dispensary and private practice, I find that I have recorded the condition of the prepuce in one hundred and fifty-three boys under nine years of age. For convenience I have classified them as follows:

| | Number. | Percentage. |
|---|---|---|
| I. Cases permitting easy and perfect retraction of the prepuce | 30 | 19.62 |
| II. Cases of slight or partial adhesion, with little or no retained smegma | 48 | 31.37 |
| III. Cases of complete or nearly complete adhesion, without stenosis, but with retained smegma, of course | 36 | 23.53 |
| IV. Cases of preputial stenosis, in which retraction was out of the question, and adhesions only to be detected by using a probe | 39 | 25.48 |
| | 153 | 100.00 |

It will be understood that the ages of those examined varied within considerable limits—some of them being infants at whose accouchment I had assisted; and still I think that my percentages will not be found far out of the way. Assuming that they represent the general average, we glean the information that only one young boy in five can easily and completely retract the prepuce. Observation confined entirely to children in the higher, or those of the lower, walks of life, may seem to make this statement incorrect; but mine is an average of all cases.

Further than this, and lending additional plausibility to my remarks in the first part of this paper, I found that of the *infants* I examined, nearly all who did not belong to Class I, did belong to Class IV; in fact they were about evenly divided between the two classes. This would seem to indicate that we must consider adhesions among the *acquired* forms of phymosis, so to speak. I have already remarked that the prepuce is usu ally long at birth. But we may draw a legitimate inference from the above that if properly directed efforts are made to date from the time when the infant receives its first bath, there would then be little difficulty in properly exposing the glans and cleaning the parts every day, and no excuse for negligence about the matter.

If preputial stenosis really do exist, the prepuce should be either slit along the dorsum, taking care to go back of the corona, the corners well rounded off and fine interrupted sutures used; or the ordinary operation of circumcision may be made. In either case any tendency to adhesion of raw surfaces may be prevented by the use of oil or some light dressing, while for the outside dressing cold water is perhaps the best.

Where simple adhesions exist a little judicious force and a little dissection by scratching with the point of a fine probe or knitting needle will be all-sufficient. For this anæsthetics are not required; for incision and stitching it is well to use them. It is well to do whatever is done as early in life as possible, for, even if nothing worse happens, stunted development of the parts may be the result of the delay.

In a number of the lads included in my second and third classes I liberated the prepuce as just described, with more or less advantage in many cases. In some it relieved the nocturnal enuresis; others were taught to regard it as a punishment for their incipient tendency to habits of self abuse; and I am happy to say in most of these latter cases it proved effectual. And in many of the cases of Class IV, wherever it was feasible, I either slit up the dorsum, or circumcised.

Paralysis and convulsions are the lesions most commonly referred to genital irritation. I have never happened to see a case of paralysis in infant or boy which I could so refer; but I have not only seen but relieved by appropriate measures infantile convulsions, general incoördination of the movements of the lower extremities, incontinence of urine, excessive irritability and nervousness, a condition of frequent semi-priapism, indigestion accompanied by a sympathetic papular eruption, chronic diarrhœa, and stunted development of the genitals; all of which could only be ascribed, after careful study, to irritation caused by one or other of the congenital or acquired forms of the lesions I have mentioned.

I doubt whether pronounced imbecility can be regarded as a reflex phenomenon of this kind; but I am sure the mental condition of all these children was improved *pari passu* with their general health. In the *N. Y. Med. Record*, April 6, 1878, there is an account of a visit made by Dr. Sayre and others to the Idiot Asylum on Randall's Island, during which visit he carefully examined the external genitals of sixty-seven of the children, operating on a number of them. While he undoubtedly benefited some of them in a way easily recognized, there is no evidence going to show that any genital lesions from which they happened to be suffering had a causative relation to their imbecility.

Of the one hundred and fifty-three little boys whom I examined, a number suffered from inguinal hernia; just *how* many I am unable to state, but, I am sure, at least twenty. My attention was not attracted by this coincidence at first so that I did not note my cases, in this respect, as carefully as I should have done. But I am sure the larger proportion of those thus affected were ranked among Class IV in my enumeration. Indeed I have never seen more than one or two children who could be placed in Class I who had any form of rupture. It is not difficult to understand how the muscular effort required to force the urine through a stenosed preputial orifice, when frequently repeated, month after month, should be sufficient to gradually cause a hernial protrusion, particularly if the parts be congenitally weak. Or it may occur before the canal (corresponding to the canal of Nuck, in the female) is completely occluded, and thus cause what is to all intents and purposes a congenital inguinal or scrotal hernia. This seems to me by far the most rational explanation of most if not all of the cases I have seen.

The possibility of diabetes mellitus occurring as the reflex result of phymosis has been broached. While I have seen one case of long standing glycosuria in a young man affected with congenital phymosis, I do not feel justified in drawing any conclusions from it, because he declined the operation. At the recent meeting of the American "Medical Association" (New York, June, 1880), Dr. Maxwell, of Delaware, reported the case of a boy, five years old, suffering from phymosis and diabetes. He circumcised the little patient and all traces of sugar quickly disappeared from the urine. This report was made in the discussion which followed Dr. G. M. Beard's paper on "Phymosis as a Cause of Nervous Symptoms," etc. Dr. Beard detailed a long list of neurasthenic symptoms in addition to those previously mentioned by other writers. These, he said, might be caused or aggravated by phymosis, or the presence of this condition might interfere with their cure. There is a hint thrown out in these last few words of whose truth my own experience has convinced me. It is that while phymosis and allied conditions may not necessarily be active exciting causes of nervous trouble, they may yet be passive obstacles to recovery from general or purely nervous disorders.

Dr. Beard also took the ground that when the nerves seemed exhausted, and the system debilitated, then local irritation might become the cause of nervous symptoms which would not otherwise have developed. He also laid stress on the reminder that immediate or startling results were not to be expected from operative treatment, the justness of which will be obvious to all; and, furthermore, that accessory and supplementary treatment may, at times, be needed. In his own practice out of eighty cases of general and sexual neurasthenia, thirty one—over one-third—had either phymosis or adherent and redundant prepuce. This, moreover, among adults.

In considering this subject one other feature has occurred to me, and that is, the practicability of doing circumcision or some similar operation as a revulsive measure, or with a view of correcting an abnormality and at the same time establishing a temporary but pronounced counter-irritation, in cases not seeming to especially demand it. More than one surgeon and neurologist has advised circumcision in idiots, imbeciles and the insane. Any suggestion to this effect would, however, be swallowed up in the general principle that no boy ought to be allowed to grow up with any abnormal condition of the prepuce. If general experience has not already shown the fundamental importance of this principle, the time is not far distant, in my opinion, when it will be clearly shown.

It is not necessary to recapitulate the disadvantages of an abnormal prepuce; they have already been considered at sufficient length. But a very brief reference to the advantage of a normal one may be allowed. In the first place it is undoubtedly the condition intended by nature, and is evidently the standard aimed at, judging from a majority of specimens of adult life. Second, a fertile cause of nervous irritation is avoided. Third, infection from venereal disease is certainly more probable where perfect cleanliness is impossible, and, *per contra*, when the parts can be thoroughly cleaned infection is much less likely to happen. Fourth, if venereal disease do exist, as for example, chancroid or gonorrhœal balanitis, it is of gravity in proportion as its site is concealed; when the diseased portion can be exposed it can be much more satisfactorily treated. Lastly, lack of cleanliness is said to be a predisposing cause of epithelioma.

The more, therefore, we consider the disadvantages of the abnormal condition and the advantages of the normal one, the more particular we should be to give our little patients the benefit of our knowledge. And every parent who has his child's best interest at heart can be soon made to see the reason of it and second the physician's efforts.

I will conclude by a very brief report of two cases referred to in the body of this paper: Two little boys under my care, one three, and the other five years of age, had both suffered for months from chronic diarrhœa. The former, the younger, when but ten months old had been operated on by a New York surgeon of note for the relief of a congenital phymosis. By the neglect of a careless mother he relapsed into as bad a condition as ever, having preputial stenosis and complete adhesions. He was excessively nervous and peevish. Although his diet was restricted his diarrhœa was only partially checked by medicines, never completely relieved. The other lad had a congenital phymosis with complete adhesions. His appetite was ravenous and his food usually passed through but little changed. Medicine seemed to do no good. He was emaciated, irritable, and suffered from prolapsus recti.

About the same operation was made in either case, to restore the normal relations of the parts. Both speedily improved in every respect. The former I saw several times, the latter I heard from; and in both the good results seemed permanent.

There are now so many cases of cure of infantile convulsions by circumcision on record, that I do not think it worth while to report any in addition. Nor would I be understood as hinting that phymosis is a common cause of diarrhœa or the other disorders I have mentioned; I simply have introduced these two cases as samples, exemplifying what a variety of lesions genital irritation may produce, and showing the advantage and the necessity of always examining the genital organs of young children, no matter what may be the malady we are called upon to treat.

1558 WABASH AVE.

